# Safety of P28GST, a Protein Derived from a Schistosome Helminth Parasite, in Patients with Crohn’s Disease: A Pilot Study (ACROHNEM)

**DOI:** 10.3390/jcm9010041

**Published:** 2019-12-24

**Authors:** Monique Capron, Laurent Béghin, Céline Leclercq, Julien Labreuche, Arnaud Dendooven, Annie Standaert, Marie Delbeke, Adeline Porcherie, Maria Nachury, Arnaud Boruchowicz, Jean-Louis Dupas, Mathurin Fumery, Thierry Paupard, Sylviane Catteau, Dominique Deplanque, Jean-Frederic Colombel, Pierre Desreumaux

**Affiliations:** 1 U995–LIRIC, Lille Inflammation Research International Center, Univ. Lille, Inserm, CHU Lille, F-59000 Lille, France; laurent.BEGHIN@laposte.net (L.B.); arnaud.dendooven@gmail.com (A.D.); annie.standaert@univ-lille.fr (A.S.); marie.delbeke@inserm.fr (M.D.); maria.nachury@chru-lille.fr (M.N.); pdesreumaux@hotmail.com (P.D.); 2 CIC 1403, Centre d’Investigation Clinique, Univ. Lille, Inserm, CHU Lille, F-59000 Lille, France; dominique.deplanque@chru-lille.fr; 3 Direction de la Recherche et de l’Innovation, CHU Lille, F-59000 Lille, France; celine.leclercq@chru-lille.fr; 4 EA 2694–Santé Publique: épidémiologie et Qualité des Soins, Univ. Lille, CHU Lille, F-59000 Lille, France; julien.labreuche@chru-lille.fr; 5 Par’Immune, Eurasante, F-59120 Loos lez Lille, France; a.porcherie@parimmune.com; 6 Service des Maladies de l’Appareil Digestif, Unité de Recherche Clinique, Centre Hospitalier de Valenciennes, F-59300 Valenciennes, France; boruchowicz-a@ch-valenciennes.fr; 7 Service d’Hépato-Gastro-Entérologie, CHU Amiens Picardie, F-80054 Amiens, France; jean-louis.dupas@gmail.com (J.-L.D.); mathurinfumery@gmail.com (M.F.); 8 Service d’Hépato-Gastro-Entérologie, Unité de Recherche Clinique, Centre Hospitalier de Dunkerque, F-59385 Dunkerque, France; thierry.paupard@ch-dunkerque.fr; 9 Service d’Hépato-Gastro-Entérologie, Unité de Recherche Clinique, Centre Hospitalier de Boulogne sur Mer, F-62200 Boulogne Sur Mer, France; sylviane.catteau@wanadoo.fr; 10 Icahn School of Medicine at Mount Sinai, New York, NY 10029, USA; jean-frederic.colombel@mssm.edu

**Keywords:** helminth protein, P28GST, Crohn’s disease, safety, Crohn’s disease activity index, calprotectin

## Abstract

Despite the development of novel therapies, inflammatory bowel diseases remain an innovative treatment challenge. Helminth therapy is a new promising approach, and a key issue is the identification of helminth-derived anti-inflammatory mediators. P28 glutathione-S-transferase (P28GST), a protein derived from schistosomes, a trematode parasitic helminth, was shown to reduce intestinal inflammation in experimental colitis by down-regulating the Th1/Th17 response. In this multicenter, open-label, pilot Phase 2a study, we evaluated the safety of P28GST administered to patients with mild Crohn’s disease (CD). We enrolled 10 patients with a baseline Crohn’s disease activity index (CDAI) value <220. Eight patients received two to three subcutaneous injections of recombinant P28GST with adjuvant. This three-month treatment was followed by a nine-month monitoring period. The primary endpoints were the monthly rate and seriousness of adverse events (AEs). Secondary endpoints were clinical recurrence, assessed with the CDAI as well as the levels of immunologic and inflammatory blood and tissue markers. The most common AEs were local or regional events at the injection site and gastrointestinal disorders. At three months after the first injection, CDAI scores and blood calprotectin levels decreased in parallel. These results indicate that P28GST showed promise as a safe and new therapeutic option for treating CD.

## 1. Introduction

Crohn’s disease (CD) is recognized as a chronic auto-inflammatory disorder of the gastrointestinal tract, which has a significant impact on patient quality of life [1]. Novel therapies are available, but they have several limitations, including lack of response, resistance development, secondary effects, and high cost. Thus, there is a persistent, major, unmet need for novel therapeutic agents with different mechanisms of action.

Epidemiologic arguments have suggested that helminths and microbiota participate in the “hygiene hypothesis” [2]. That hypothesis holds that helminth parasites play the role of “old friends”, which counterbalance the overactive Th1/Th17 response. A recent study reviewed the role of helminths in the balance between immunoregulatory and effector mechanisms [3].

Animal models of colitis revealed that helminths could produce immunomodulatory substances that inhibited intestinal inflammation. Both the ES-62 glycoprotein from the filarial nematode, *Acanthocheilonema vitae*, and the P28 glutathione-S-transferase (P28GST) protein from schistosomes could decrease the inflammatory response (Th1 and Th17) and induce an anti-inflammatory/immuno-regulatory response (Th2/Treg). Those findings led to the concept that well-defined products derived from helminths could be used as anti-inflammatory drugs in place of the entire parasite [4]. 

P28GST, an enzymatic protein derived from *Schistosoma haematobium* (Sh28GST), induced an IL-10 response mediated by Th2/T-regulatory cells (Tregs), in several animal species, including human [5,6]. P28GST was first developed as a vaccine against schistosomiasis. In that context, P28GST demonstrated safety and tolerability, both in adults [6] and children (NCT 00870649 [7]). P28GST was also tested in experimental colitis, where it reduced colonic inflammation. In these studies, alum adjuvant was shown to be required as a potent stimulus of the Th2 response. Based on those findings, P28GST has been repositioned as a potent anti-inflammatory drug in auto-inflammatory–autoimmune disorders, such as CD. 

The P28GST-driven reduction in colonic inflammation was associated with a reduction in the Th1/Th17 inflammatory response, and an increase in the immunoregulatory response involving Th2/Treg cells and M2 macrophages [5,8,9]. Specifically, P28GST combines the antigenic properties and regulatory enzymatic activities of the antioxidant GST with the anti-inflammatory properties of prostaglandin-D-synthase. According to the “equilibrium model of immunity,” by inducing the Th2 response, P28GST inhibits autoimmune Type 3 inflammation [3].

Here, we conducted a pilot Phase 2a clinical study to evaluate the safety of P28GST treatment in patients with mild CD.

## 2. Materials and Methods 

This study was approved by the French Health Authority (Agence Nationale de Sécurité des Médicaments, ANSM) and the France Nord-Ouest IV Ethics Committee. It was also approved by the European Medicines Agency (EudraCT number 2013-000595-15). This study was conducted in accordance with the Declaration of Helsinki, and it complied with the International Conference on Harmonization Good Clinical Guidelines. All patients provided written informed consent for participation before study inclusion. Trial Registration: NCT02281916.

### 2.1. Study Design 

**Figure 1:**
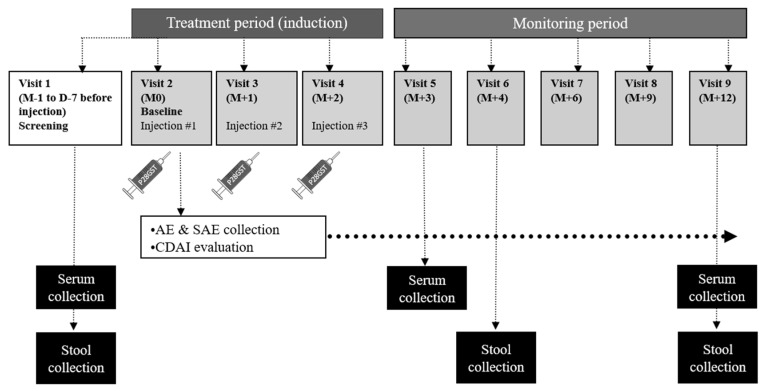
Open-label study design for evaluating the safety of P28GST treatment in patients with mild Crohn’s disease. The time of each hospital visit is given by the number of months (M) or days (D) before (−) or after (+) the first injection (injection #1 at M0). AE: adverse event; SAE: serious adverse event; CDAI: Crohn’s disease activity index.

This multicenter, one-year study included a three-month treatment period, followed by a nine-month monitoring follow-up (ClinicalTrials.gov: NCT02281916; Figure 1). This study was sponsored by the Lille University Hospital (Lille, France). It was conducted at five centers in Northern France, between 2014 and 2018. 

The main inclusion criteria were: (i) a minimum age of 18 years, (ii) mild CD, and (iii) a CDAI score <220. The main exclusion criteria were: the use of tobacco; the use of azathioprine, anti-tumor necrosis factor, methotrexate, Vedolizumab, Ustekinumab, or other immunosuppressors, within eight weeks prior to the first P28GST injection; the use of corticosteroids within the 15 days prior to the first P28GST injection; and a history of vaccine hypersensitivity or allergy, AIDS, hepatitis B or C, or any other clinical manifestation detected by the investigator.

### 2.2. Experimental Treatment

The P28GST protein was produced as a recombinant protein (rSh28GST) from *Saccharomyces cerevisiae* cultures (TGY73.4-pTG8889 strain). The manufacturing process was conducted according to good manufacturing practice (GMP) guidelines, by Eurogentec S.A. (Seraing, Belgium), as described previously [7]. The rSh28GST clinical batch (M-BIX-P03/225a) was lyophilized under GMP conditions by Miltenyi (Bergisch Gladbach, Germany), with 225 µg (±10%) per vial. Before patient administration, the lyophilized preparation was re-suspended extemporaneously in 1 mL of apyrogenic, sterile 0.2% alhydrogel solution (0.2% Al_2_O_3_; 3% Al(OH)^3^; 9 g/L NaCl; 10 mM ammonium carbonate buffer, pH 7.8; batch #P16-14-01; Bio Elpida, Saint Priest, France) in sterile conditions.

Each patient received two to three subcutaneous injections (0.4 mL per injection) of recombinant rSh28GST, for a total administration of 100 µg per injection. The injections were administered over two to three months [6] (Figure 1: Open-label study design).

### 2.3. Safety Outcomes 

The primary objective was to evaluate the tolerance to P28GST treatment, formulated with the adjuvant, alum. Safety outcomes included the number and intensity of adverse events (AEs), which were classified as the following types: AEs of special interest (AESIs), other AEs, and serious AEs (SAEs). AESIs were defined as the AEs likely to occur with P28GST treatment. We defined three types of AESIs: clinical and paraclinical events (events associated with Crohn’s disease, abnormal electrocardiogram results, increased body temperature, or sleep disorders); loco-regional events at the site of injection (such as skin reactions, hypoaesthesia, pain, erythema); and biological events (such as hematological abnormalities, liver and renal dysfunctions, and abnormal levels of blood creatinine, phosphokinase, lipase, or lactate dehydrogenase, detected in a standard blood test). The other AEs were classified, based on a Medical Dictionary for Regulatory Activities (MedDRA, version 21.1). All AEs were graded by intensity as mild, moderate, severe, or life-threatening. The causal relationships between AEs and P28GST treatment were established according to the method of Bégaud et al. [10], and classified as follows: not related, possibly related, probably related, and certainly related. SAEs that were suspected to be related to P28GST treatment, but never previously described, were called suspected, unexpected serious adverse reactions (SUSARs) [6,7]. Safety outcomes were assessed at baseline (pre-inclusion visit), at inclusion, and at months 1, 2, 3, 4, 6, 9, and 12. The safety follow-up visit at month 12 represented the end of the study. A Data Safety Monitoring Board was set up with four independent expert members to perform regular safety data analyses.

### 2.4. Secondary Outcomes

The Lille University Hospital, as sponsor of this assay, has taken in charge the standardization of the results, in its dedicated “data management” structure and the results have been audited by an external independent society.

To complement the safety criteria for P28GST treatment, we also analyzed CDAI scores, serum calprotectin (SC), fecal calprotectin (FC), C-reactive protein (CRP), and specific antibodies to P28GST. We calculated CDAI scores, based on diary entries recorded by the patient over seven consecutive days prior to each hospital visit [11].

Fecal samples were collected by patients at home, before treatment, and at 4 and 12 months after the first injection. On the morning day of sample collection, each patient collected a pellet (~20 g) of feces using a fecal collector kit containing: a feces catcher Bepan Liner (Carebag, Cleanis©, Arcueil, France), a sterile stool collection tube with scoop, and gloves. Samples were immediately stored at 4 °C in the home fridge and were transported as soon as possible (maximum time delivery: 6 h) in an isothermic bag at 4 °C to the Biological Center of Lille University Hospital (CIC/CRB 1403, Inserm-CHU de Lille) or the hospital laboratory (for study locations other than Lille University Hospital). Then, samples were aliquoted (two tubes: one for the calprotectin measurement and the other one for microbiota analysis) and stored at −80 °C for further analysis.

FC was measured in the central laboratory of Lille University Hospital, with a commercially available enzyme-linked immunoassay (ELISA) kit (EK-CAL, Bühlmann, Switzerland), according to the manufacturer instructions [12]. FC levels are expressed as μg/g of feces, and the normal range is FC < 50 µg/g.

Blood samples were collected by venous puncture, and sera were stored at −80 °C until analysis. SC was measured in the central laboratory of Lille University Hospital, with a commercially available ELISA kit (EK-MRP8/14, Bühlmann, Switzerland), according to manufacturer instructions. SC levels are expressed as μg/mL of serum (normal value: <2 µg/mL; weakly positive: >2–4 µg/mL: and positive: >4 µg/mL). Serum CRP and other laboratory markers were routinely analyzed in each hospital laboratory (normal values ranged between 1 and 8 µg/mL). 

Serum antibodies specific to P28GST were measured in all patients with ELISA kits, according to Riveau et al. [7]. Results are presented as the individual titers at baseline and at 3 and 12 months after the first P28GST injection. Briefly, rSh28GST (10 µg/mL) was coated on 96 well plates (Nunc, Roskilde, Vaud, Denmark) for 2 h 30 min at 37 °C. Plates were blocked with phosphate-buffered saline containing 0.5% gelatin (Merck, Darmstadt, Hesse, Germany), then serial dilutions of individual sera were added and incubated overnight at 4 °C. Biotinylated monoclonal antibodies specific for different human Ig isotypes (BD Pharmingen) were added for 1 h 30 min at 37 °C. The dilutions were: 1/2000 for total IgG, 1/500 for IgE, and 1/3000 for IgA1/A2. Peroxidase-conjugated streptavidin (diluted 1/20,000; BD Pharmingen) was added for 30 min at 37 °C. Bound secondary antibodies to IgG, IgE, and IgA were quantified with the ABTS substrate (Sigma, Aldrich, Merck, Darmstadt, Germany). Optical densities were evaluated at 450 nm (Fluostar Omega, BMG). Titers were defined as the highest dilution to yield an absorbance two-fold above the negative control.

Cytokine levels in sera were measured with a Luminex assay, as recommended by the manufacturer (Bio-Techne, Minneapolis, MN, USA).

### 2.5. Statistical Analyses

Quantitative variables are expressed as the median (range). Baseline CDAI scores, SC levels, and CRP levels were compared to the 3- and 12-month post-treatment values with Wilcoxon signed-rank tests. The 3- and 12-month changes were expressed as the median % decrease from the baseline value. Statistical testing was performed at the 2-tailed α-level of 0.05. Data were analyzed with SAS software (version 9.4, SAS Institute Inc., Cary, NC, USA). 

## 3. Results

### 3.1. Patients 

This study included a total of 10 patients that fulfilled the inclusion/exclusion criteria. Of these 10 patients, 2 discontinued participation early, before the first P28GST injection; of these, one failed the screening process and the other withdrew consent. Thus, a total of 8 patients (5 males, 3 females) were treated with P28GST. Among these 8 patients, 2 received only two P28GST injections instead of the planned three injections. 

As shown in Table 1, the 8 included patients had mild or inactive CD. The mean CDAI score was 96 (11–178).

### 3.2. Safety Outcomes

During the study, all treated patients reported at least one AE, and a total of 39 AEs occurred. Among these, 25 were certainly or possibly related to P28GST treatment, including 12 AESIs and 13 other AEs (Table 2). All AEs were graded as mild or moderate. 

All AESIs were due to loco-regional events at the injection site (Table 3), except for one adverse event (sleep disorder). All AESIs were resolved. Three patients reported local injection-site events that were certainly related to P28GST treatment. These patients experienced pain, hypoaesthesia, erythema, and reactions at the injection site. Of the 11 loco-regional events at the injection site, 7 were observed in a single patient and occurred after each P28GST injection (Table 3). That patient discontinued P28GST treatment and the follow-up study after the second P28GST injection, due to a tetanus vaccination, which was contraindicated with P28GST treatment for this study.

Four patients reported 13 other AEs that were possibly related to P28GST treatment (Table 4). Of these, one patient experienced a single AE: Verneuil’s disease (termed hidradenitis, in MedDRA). Most of the other AEs occurred in only 2 patients; each reported 5 AEs. In both these patients, the gastrointestinal events (abdominal pain, diarrhea, and vomiting) spontaneously resolved, and blurred vision spontaneously resolved. One of these 2 patients reported hematospermia with polyuria without any other urinary dysfunction signs; this condition also resolved without specific treatment. This patient was the only one with 2 SAEs that were considered SUSARs, and both SUSARs were classified as “possibly related” to P28GST treatment.

The first SUSAR was vestibular neuronitis, which was reported 8 days after the second P28GST injection, and it led to a premature discontinuation of P28GST treatment. This condition was accompanied by an inflammatory syndrome, with an underlying ear, nose, and throat infection, and hypergammaglobulinemia. The vestibular neuronitis resolved after 1.5 months, with corticoids and anti-inflammatory drug treatment. The second SUSAR, which occurred in the same patient, was renal failure, probably functional. This SUSAR occurred 5 months after the second P28GST injection. However, it was not examined further, because it spontaneously resolved within 8 days (Table 4). 

Only one premature discontinuation of P28GST treatment was related to an SAE occurrence. This patient did not receive the third injection of P28GST. However, this patient accepted to continue the follow-up despite not receiving the third injection.

### 3.3. Secondary Outcomes 

To evaluate the safety of P28GST treatment further, we analyzed the individual CDAI scores, SC and CRP levels at baseline and at 3 and 12 months after treatment. We also analyzed the levels of specific P28GST antibodies. Due to an AE unrelated to the treatment, which led to premature discontinuation, one patient was not included in the following analyses.

**Figure 2:**
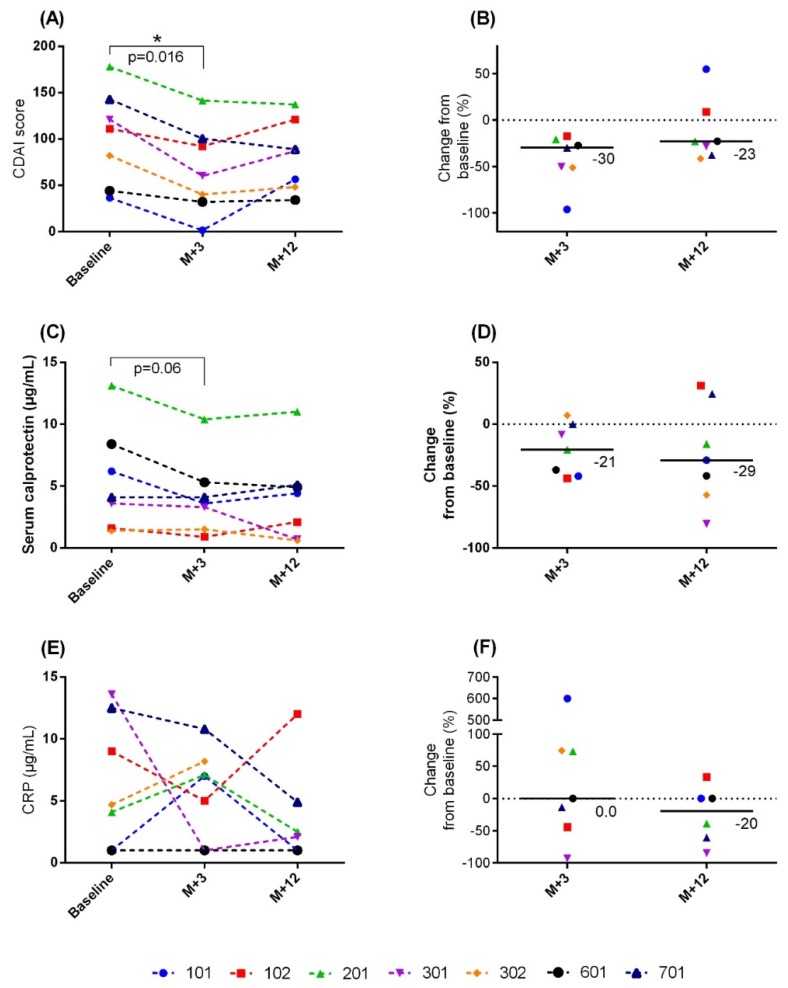
Safety of P28GST treatment, based on individual scores and inflammation markers. (**A**) CDAI scores; (**B**) CDAI change from baseline; (**C**) SC levels; (**D**) SC change from baseline; (**E**) CRP levels; (**F**) CRP change from baseline. Individual patients are identified by ID code numbers, which are color-coded; follow-ups were performed at 3 months (M + 3) and 12 months (M + 12) after the first P28GST injection. CDAI: Crohn’s disease activity index. *: *p* < 0.05.

The CDAI scores significantly decreased at 3 months compared to baseline (Figure 2A), for all seven patients with available post-treatment scores (*p* = 0.016). The corresponding median relative decrease from baseline was 30% (range, −96% to −17%; Figure 2B). At 9 months, 5 of 7 patients had CDAI scores lower than baseline. The median relative decrease from baseline was 23% (range, −41% to 55%; Figure 2A,B). At 12 months, the difference between baseline and post-treatment values did not reach a significant level. 

The SC levels decreased at 3 months and 12 months in 5 of the 7 treated patients. In the other 2 patients, the SC levels remained stable. However, these differences did not achieve significance (*p* = 0.06). The SC levels improved with the CDAI scores at 3 months and 12 months (Figure 2C). The median relative decreases in SC from baseline were 21% (range, −44% to 7%) at 3 months and 29% (range, −81% to 31%) at 12 months (Figure 2D).

The median baseline FC was 312 µg/g (range: 75–678). Although it is difficult to interpret these results, due to missing samples, the FC levels decreased after treatment in 4 out of the 6 evaluable patients (data not shown).

The serum CRP levels decreased more slowly than the SC levels (Figure 2E,F). The median relative decrease in CRP from baseline was 20% (range, −85% to 33%) at 12 months. However, the difference between post-treatment values and baseline did not reach significance. 

### 3.4. Immunological Analysis

**Figure 3:**
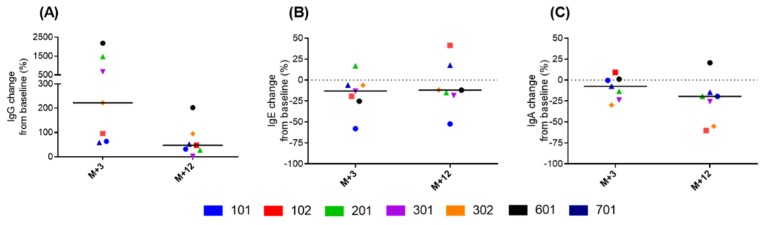
Changes in specific anti-P28GST antibody levels after treatment. Changes in anti-P28GST (**A**) IgG, (**B**) IgE, and (**C**) IgA antibodies from baseline are shown for each patient at 3 months (M + 3) and 12 months (M + 12) after the first P28GST injection. Individual patients are identified by numbers, which are color-coded.

Specific anti-P28GST IgG, IgE, and IgA antibodies were measured at 3 and 12 months after the first P28GST injection (Figure 3). In all patients, IgG antibodies increased from baseline at 3 months after the first injection (between 100% and 2000% of baseline). Then, IgG levels decreased at the end of the monitoring period. In contrast, anti-P28GST IgE and IgA antibodies were lower than baseline after treatment, at the end of the study 

We also measured serum levels of cytokines, chemokines, and growth factors with Multiplex technology in all patients. We observed some changes in cytokine levels after P28GST treatment. However, our results were consistent with those from previous studies, which demonstrated the infeasibility of considering circulating cytokines as biomarkers for inflammatory bowel diseases (IBD) [13]. 

## 4. Discussion 

To the best of our knowledge, this pilot study was the first clinical trial to evaluate the safety of a recombinant helminth protein in patients with CD. We investigated P28GST-related AEs in patients with mild or inactive CD. Although our study sample was limited, we aimed to refine the critical elements of safety for use in a future, randomized, controlled trial, which will test the ability of a helminth protein treatment to modulate inflammation in IBD. Our findings revealed that P28GST treatment was sufficiently tolerable for these CD patients.

Pioneering clinical trials showed that treatments of helminth eggs or larvae provided some clinical improvement in patients with CD and ulcerative colitis with no AEs [4,14,15]. However, a recent phase 2, randomized, placebo-controlled, multicenter trial that tested *Trichuris suis* ova (TSO) in patients with active CD showed that TSO efficacy was not significantly superior to placebo [16]. In addition to testing helminths as an alternative therapy for intestinal diseases, helminth-derived anti-inflammatory mediators have been identified. These mediators represent a more promising approach because they can replicate the benefits without the detriments of live helminths [16].

The primary objective of the present pilot study was to determine the safety of a helminth-derived protein for treating mild or inactive CD. This protein was found to be safe in previous trials [6,7]. We found a total of 25 AEs that were related or possibly related to the treatment. Loco-regional events at the injection site contributed to almost all the AESIs. These local injection site events, which occurred in only 3 out of the 8 patients, were expected, because they were also reported in a previous trial that used the same protocol for treating a different disease [7]. All the AESIs resolved completely with time. Two SAEs, classified as possibly related to the treatment, were not expected, and these were defined as SUSARs. These two SUSARs appeared in succession in the same patient (101) after the second injection. One SUSAR was vestibular neuronitis (which resolved with anti-inflammatory treatment) and the other was a renal manifestation (which spontaneously resolved). The protocol was interrupted (no third injection), but the patient completed the follow-up until the end of the protocol. 

Vestibular neuronitis is typically linked to an acute inflammatory state, accompanied by elevated blood CRP. However, in our patient, vestibular neuronitis was accompanied by a very moderate and transient increase in blood CRP at the time of the AE occurrence. It is interesting to notice that the ear is a rare but recognized site of extra-intestinal manifestations of IBD [17]. The etiology of vestibular neuronitis disease remains unclear. It is typically considered a benign condition, linked to an infection that originally affected the upper respiratory tract (e.g., herpes, influenza, Epstein–Barr virus) [18]. Other potential pathogenic pathways include vascular disorders; this potential pathogenesis was supported by the presence of a pro-inflammatory state and the sudden upset of the disease. Another hypothesis holds that vestibular neuronitis might be an autoimmune disease, but further investigation in our patient did not confirm this hypothesis. Treatment with corticosteroids brought about the total recovery of peripheral vestibular functions.

It should be noted that, although the investigator classified Verneuil’s disease (*Hidradenitis suppurativa* (HS)) as “possibly” linked to treatment, this disease was previously shown to be associated with CD in 17% of patients [19]. Those authors suggested that the link between HS and CD could be stronger than expected, because HS is typically underdiagnosed in the IBD population [20].

One of the largest challenges faced in this trial was participant recruitment, which is commonly among the most challenging aspects of study executions [21]. The initial primary objective, planned in 2013, was to evaluate the effect of P28GST administered to patients with CD shortly after surgery. The initial aim was to evaluate the potential of P28GST to reduce the risk of endoscopic recurrence, which was estimated to be 70%. Although each center in the study initially agreed to recruit 4 patients per year, this number could not be achieved. Thus, after approval from the Scientific Council and the regulatory agencies, the protocol was amended in 2016 to include patients with mild CD and CDAIs <220, with or without surgery. The recruitment took longer to complete than anticipated; consequently, additional stability tests were required. Nevertheless, this pilot study was not interrupted, and the results were encouraging, though the quantitative analysis was limited. Moreover, this study provided an opportunity to monitor the stability of P28GST at regular time intervals.

As additional markers of safety, CDAI scores and SC levels were evaluated at baseline and at 3 and 12 months after the beginning of treatment. All treated patients with available post-treatment scores showed a significant, 30% reduction in the median CDAI score compared to baseline, at 3 months after the first injection of P28GST. Moreover, a highly significant reduction was observed for all patients (*p* = 0.016). To circumvent a potential placebo effect, a likely hypothesis in the absence of control group, these results were consolidated by measuring calprotectin. Although FC is the accepted marker of short-term clinical outcomes in IBD [12], we recognized that collecting stool samples was a hurdle for the patients, which could lead to many missing stool samples [22]. Therefore, we evaluated SC levels, and the results showed that SC levels decreased in parallel with CDAI scores at 3 and 12 months after P28GST treatment. Calprotectin synthesis occurs in activated inflammatory cells, including neutrophils and macrophages. These cells were targeted by the anti-inflammatory properties of P28GST [5,8]. In contrast, the total serum CRP levels did not change in parallel with the CDAI values or SC levels, at either 3 or 12 months after treatment. This result was not consistent with previous results, which demonstrated the high sensitivity of CRP in CD [22]. Nevertheless, we noted a change from baseline CRP that reached −20% at 12 months. Although our data were limited, the reduction in FC observed in 4 out of 6 patients was encouraging. Although these results were limited by the low CDAI scores at inclusion, they provided additional information about the safety of P28GST treatment, i.e., P28GST did not increase the scores or the studied markers. The requirement for adjuvant could not be evaluated in this pilot study, but non-clinical recent results indicate that subcutaneous injection of P28GST in the presence of alum induced the recruitment of regulatory dendritic cells, as well as the major role of IL-10 and M2 anti-inflammatory macrophages (unpublished results) 

We observed a specific IgG response shortly after treatment, which decreased at the end of the monitoring period. Interestingly, the specific IgE response was limited, which suggested that P28GST was associated with a low risk of developing a deleterious allergic response. Similarly, we detected low levels of IgA antibodies, which were identified as neutralizing antibodies, in these patients with CD after P28GST treatment. Taken together, these results were comparable to our previous findings in children with schistosome infections. In that study, too, after the P28GST treatment, the IgG levels increased; IgE levels increased only slightly, and IgA levels did not increase [7]. Finally, we detected no significant changes in gut microbiota composition, as reported previously [9].

## 5. Conclusions

In summary, this pilot phase 2a study showed that, among patients with mild CD, P28GST treatment was rather safe. The various scores and blood markers tested showed no increases with treatment. Because this investigation was a pilot study, these findings should not be over-interpreted; however, our preliminary data indicated that P28GST was safe and tolerable in patients with mild CD and shows therapeutic potential. Several questions remain to be investigated regarding the use of P28GST in intestinal diseases, such as: what are the appropriate dosing regimens? And, what is the optimal timing for treatment? Moreover, we lack elucidation of the exact mechanisms that give rise to the protective effects of P28GST. Fully powered clinical studies with adequate sample sizes and randomization are required to validate these results. Moreover, future studies should extend the patient population to include patients with more severe CD and ulcerative colitis.

## Figures and Tables

**Table 1:** Baseline characteristics of patients with mild Crohn’s disease.

Anthropometric Characteristics (*n* = 8)	Median (range)
Age (years)	32 (22–44)
BMI (kg/m^2^)	24 (20–29)
**Crohn’s disease indicators** (*n* = 8)	
Age of onset (years)	22 (16–35)
Disease duration (months)	7 (2–16)
CDAI	96 (11–178)

CDAI: Crohn’s disease Activity Index. BMI: Body Mass Index.

**Table 2:** All AEs that occurred during and after treatment, until the 12-month follow-up.

Events	Number of Patients	Number of AEs
Treatment-Emergent AEs, All Causalities *		
**AESI**	4	12
Not related to the study	0	0
Possibly related to the study	1	1
Probably related to the study	0	0
Certainly related to the study	3	11
**Other AE**	8	27
Not related to the study	7	14
Possibly related to the study	4	13
Probably related to the study	0	0
Certainly related to the study	0	0
**SAE**	1	2
Not related to the study	0	0
Possibly related to the study	1	2
Probably related to the study	0	0
Certainly related to the study	0	0
**Treatment-Emergent AEs that Led to Study Discontinuation**		
AESI	0	0
Other AE	0	0
SAE	1	1

* Causalities defined according to the Bégaud’s method [10]. AE: adverse event; AESI: adverse event of special interest; SAE: serious adverse event.

**Table 3:** Treatment-emergent AESIs, classified by the certainty of a causal relationship with treatment (certainly or possibly related) and the intensity of the event (grade).

	Type of Event	Grade, Specific AESIs		
		Mild	Moderate	Severe
**AESIs certainly related to the study**				
General disorders and administration site conditions	Injection site erythema	2 (1)	1 (1) *	0 (0)
	Injection site hypoaesthesia	0 (0)	2 (1) *	0 (0)
	Injection site pain	1 (1)	2 (1) *	0 (0)
	Injection site reaction	1 (1)	2 (1) *	0 (0)
**AESIs possibly related to the study**				
Psychiatric disorders	Sleep disorder	0 (0)	1 (1)	0 (0)

* The AESI occurred at each hospital visit in the same patient, and that patient discontinued treatment prematurely due to a tetanus vaccination; AESI: adverse event of special interest.

**Table 4:** Treatment-emergent other AEs and SAEs, classified by the certainty of a causal relationship with treatment (possibly related) and the intensity of the event (grade).

	Type of Event	Grade, Specific AEs (Number of Patients with AEs)		
		Mild	Moderate	Severe
**AEs possibly related to the study**				
General disorders and administration site conditions	Asthenia	1 (1)	1 (1)	0 (0)
Ear and labyrinth disorders	Vertigo	1 (1)	0 (0)	0 (0)
Eye disorders	Blurred vision	2 (2)	0 (0)	0 (0)
Gastrointestinal disorders	Abdominal pain	2 (2)	0 (0)	0 (0)
	Diarrhea	1 (1)	0 (0)	0 (0)
	Vomiting	1 (1)	0 (0)	0 (0)
Nervous system disorders	Headache	1 (1)	0 (0)	0 (0)
	Migraine	0 (0)	1 (1)	0 (0)
Reproductive system and breast disorders	Hematospermia	1 (1)	0 (0)	0 (0)
Skin and subcutaneous tissue disorders	Hidradenitis	0 (0)	1 (1)	0 (0)
**SAEs possibly related to the study**				
Ear and labyrinth disorders	Vestibular neuronitis *	0 (0)	1 (1)	0 (0)
Renal and urinary disorders	Renal failure *	0 (0)	1 (1)	0 (0)

* SAEs occurred in the same patient, who discontinued treatment prematurely due to vestibular neuronitis. AE: adverse event; SAE: serious adverse event.
